# Rare Primary Central Nervous System Tumors in Adults: An Overview

**DOI:** 10.3389/fonc.2020.00996

**Published:** 2020-06-26

**Authors:** Enrico Franceschi, Didier Frappaz, Roberta Rudà, Peter Hau, Matthias Preusser, Caroline Houillier, Giuseppe Lombardi, Sofia Asioli, Caroline Dehais, Franck Bielle, Vincenzo Di Nunno, Martin van den Bent, Alba A. Brandes, Ahmed Idbaih, EURACAN Domain 10, Paul Clement Radek

**Affiliations:** ^1^Department of Medical Oncology, Azienda USL/IRCCS Institute of Neurological Sciences, Bologna, Italy; ^2^Department of Neuro-Oncology and Institut d'Hématologie et d'Oncologie Pédiatrique, Centre Léon Bérard, Lyon, France; ^3^Department of Neuro-Oncology, City of Health and Science and University of Turin, Turin, Italy; ^4^Wilhelm Sander NeuroOncology-Unit, Department of Neurology, University Hospital Regensburg, Regensburg, Germany; ^5^Division of Oncology, Department of Medicine I, Medical University of Vienna, Vienna, Austria; ^6^Sorbonne Université, IHU, ICM, Service de Neurologie 2-Mazarin, Groupe Hospitalier Pitié-Salpêtrière, Paris, France; ^7^Department of Oncology, Veneto Institute of Oncology-IRCCS, Padua, Italy; ^8^Section of Anatomic Pathology “M. Malpighi”, Department of Biomedical and Neuromotor Sciences, Bellaria Hospital, Bologna, Italy; ^9^Sorbonne Université, Inserm, CNRS, UMR S 1127, Institut du Cerveau et de la Moelle épinière, ICM, AP-HP, Hôpitaux Universitaires La Pitié Salpêtrière - Charles Foix, Service de Neurologie 2-Mazarin, Paris, France; ^10^Department of Neuropathology, Hôpitaux Universitaires La Pitié Salpêtrière - Charles Foix, AP-HP, Sorbonne Université, SIRIC Curamus, Paris, France; ^11^The Brain Tumor Center at Erasmus MC Cancer Institute, Rotterdam, Netherlands

**Keywords:** pineal tumors, mesenchymal non meningothelial intracranial tumors, CNS lymphoma, germ cell tumors, pituitary tumor, glioneural tumor, embryonal tumor of central nervous system, medullobalstoma

## Abstract

Overall, tumors of primary central nervous system (CNS) are quite common in adults with an incidence rate close to 30 new cases/100,000 inhabitants per year. Significant clinical and biological advances have been accomplished in the most common adult primary CNS tumors (i.e., diffuse gliomas). However, most CNS tumor subtypes are rare with an incidence rate below the threshold defining rare disease of 6.0 new cases/100,000 inhabitants per year. Close to 150 entities of primary CNS tumors have now been identified by the novel integrated histomolecular classification published by the World Health Organization (WHO) and its updates by the c-IMPACT NOW consortium (the Consortium to Inform Molecular and Practical Approaches to CNS Tumor Taxonomy). While these entities can be better classified into smaller groups either by their histomolecular features and/or by their location, assessing their treatment by clinical trials and improving the survival of patients remain challenging. Despite these tumors are rare, research, and advances remain slower compared to diffuse gliomas for instance. In some cases (i.e., ependymoma, medulloblastoma) the understanding is high because single or few driver mutations have been defined. The European Union has launched European Reference Networks (ERNs) dedicated to support advances on the clinical side of rare diseases including rare cancers. The ERN for rare solid adult tumors is termed EURACAN. Within EURACAN, Domain 10 brings together the European patient advocacy groups (ePAGs) and physicians dedicated to improving outcomes in rare primary CNS tumors and also aims at supporting research, care and teaching in the field. In this review, we discuss the relevant biological and clinical characteristics, clinical management of patients, and research directions for the following types of rare primary CNS tumors: medulloblastoma, pineal region tumors, glioneuronal and rare glial tumors, ependymal tumors, grade III meningioma and mesenchymal tumors, primary central nervous system lymphoma, germ cell tumors, spinal cord tumors and rare pituitary tumors.

## Introduction

The European Union has launched 24 European Reference Networks (ERNs). ERNs are virtual networks involving healthcare providers across Europe aimed to tackle complex or rare diseases that require highly specialized approaches and concentrated knowledge and resources. The ERNs include a network for rare adult solid cancers called EURACAN.

Taken in totality as one group of cancer, primary central nervous system (CNS) tumors are quite common with an incidence rate close to 30 new cases/100000 inhabitants per year, in adults. Nevertheless, more than 150 entities—many of which are rare or ultrarare—have now been categorized by the World Health Organization and by the c-IMPACT NOW consortium (1). Each of them has an incidence rate below 6.0 new cases per year/100,000 inhabitants (which is the threshold definition for rare diseases). The most common adult primary CNS tumors are diffuse gliomas and grade I and II meningiomas.

Many other entities grouped either by ontogenesis, by histomolecular features or by CNS location are less common. These include medulloblastoma and embryonal tumors, pineal region tumors, glioneuronal and rare glial tumors, ependymal tumors, grade III meningioma and mesenchymal tumors, primary CNS lymphoma and hematological diseases, germ cell tumors, spinal cord tumors, rare pituitary tumors and ultrarare tumors. Due to their rarity, progress in biological and clinical research is in most of the cases slow.

Within EURACAN, Domain 10 (the ERN for adult rare primary CNS tumors) brings together the European patient advocacy groups (ePAGs), physicians and researchers from EU countries, who are highly specialized in the field of rare primary CNS tumors and who support research, care and teaching in the field. EURACAN is therefore working in close collaboration with European neuro-oncology groups, particularly: (i) the Brain Tumor Group of the European Organization for Research and Treatment of Cancer (EORTC BTG), (ii) the European Association of Neuro-Oncology (EANO), (iii) national neuro-oncology working groups and, (iv) and with ePAGs in particular the International Brain Tumor Alliance (IBTA).

EURACAN Domain 10 is structured in multiple subdomains to cover all subgroups of rare adult primary CNS tumors. Medical and biological advances to date are summarized in the current review.

## Medulloblastoma and Embryonal Tumors

### Medulloblastoma

Medulloblastoma is an embryonal tumor originating from cerebellum, is rare in post-pubescence and adulthood with an estimated incidence of 0.05–0.1 patients per 100,000 per year (2–9).

Medulloblastoma biology varies between different age groups, resulting in distinct tumor subtypes with distinct clinical behavior. The diagnostic and therapeutic pathways suggested for adult and post-pubertal patients in a recent European consensus paper are based largely on extrapolations of pediatric data (3).

The heterogeneity of medulloblastoma gives rise to a range of subtypes. The revised 2016 WHO classification provides a definition of medulloblastoma according to both histologic and genetic features (1). Four distinct subgroups can be distinguished at the genetic level: (i) WNT activated; (ii) SHH activated and *TP53* wildtype; (iii) SHH activated and *TP53* mutant; and (iv) non-WNT/non-SHH.

SHH-activated *TP53*-wildtype medulloblastomas are the most common in adults, representing about 60% of cases (10). Mutations in either *PTCH1* or *SMO* are present in over 80% of this subgroup in adults and represent excellent candidates for molecularly targeted therapy (11). While rare in adult SHH-driven medulloblastomas, the *TP53* mutation confers a worse prognosis (12). Adults with WNT-driven medulloblastoma constitute about 15%, and non-WNT/non-SHH medulloblastomas around 25% of cases (10).

The classical Chang staging system considers the size and amount of tumor infiltration (T1–T4) and divided metastatic stage into M0 to M4 (13). However, prognostic factors identified in trials with pediatric patients have not been unequivocally confirmed in adults. In contrast to the situation in children, the prognostic role of M stage and post-resection residual tumor >1.5 cm^2^ (9, 14) have not yet been convincingly validated.

Outcomes of the different medulloblastoma subgroups vary depending on age (10, 15). Large cell/anaplastic histology is a high-risk feature in adults (16). Whereas, adults with SHH-driven medulloblastoma have more favorable overall survival (OS) than non-WNT/non-SHH tumors (16, 17), adults with WNT-driven medulloblastoma do not show the favorable outcomes of pediatric patients (17, 18). The *TP53* mutation is associated with a poor prognosis (12, 16), while *MYCN* amplification confers a worse prognosis in the SHH subtype (16), it does not in group 4 tumors (18).

Medulloblastoma affords a curative treatment approach and thus involves intensive treatments that bear the risk of clinically relevant side effects. Gross total resection (GTR) should be performed in all patients to alleviate symptoms and facilitate diagnosis (4). The backbone of post-operative treatment with adjuvant chemoradiation comprises craniospinal irradiation (CSI) to a total dose of 36 or 35.2 Gy and local dose escalation to the posterior fossa (to a total dose up to 54–55.8 Gy) (5). Lower doses for CSI of 23.4 Gy or even 16 Gy have been systematically investigated in children, but not in adults, with comparable efficacy (19). In many centers worldwide, lower CSI doses are also used in adults. If available, proton therapy can be considered as an alternative to helical IMRT (Intensity-Modulated RadioTherapy) or VMAT (Volumetric Modulated Arc Therapy) for reduction of long-term side effects (20, 21). Similar survival outcomes were reached in children with protons compared to photon irradiation (22). Recommendations about systemic treatments are mainly provided by the results of pediatric trials, retrospective series of adult cohorts within pediatric trials with only single single-arm prospective trial in adults (6–9). It must be realized that the tolerance for chemotherapy is less in adults as compared to children.

Medulloblastomas are comparably well-understood and thus amenable to risk stratification and targeted therapies (11, 17). Stratification of patients into prognostic and therapeutic subgroups is a prerequisite to individualized treatment, e.g., to identify patients who may benefit from IMRT-chemotherapy or targeted treatment. However, prospectively validated predictors are scarce in post-pubertal and adult patients. The first genotype-based trial in adults (EORTC 1634-BTG) will start in 2020.

Due to the CNS location of medulloblastomas, late toxicity is a key issue (23). In an effort to reduce toxicity, standard CSI to a total dose of 36 or 35.2 Gy (five times weekly) can likely be reduced to 23.4 Gy without loss of efficacy if combined with chemotherapy (24) and dose escalation to the tumor bed >50 Gy (25). Packer chemotherapy is less well-tolerated by adults than children (6, 7, 26). Moreover, lower doses of the chemotherapeutic agent cisplatin do not seem to result in decreased efficacy, and strict stopping rules in cases of ototoxicity thus seem justified (27), at least in children. Substitution of cisplatin for carboplatin has not been systematically investigated in adults.

Although the data are currently limited to individual objective responses in small cohorts (28, 29) the most promising and mature targeted approach is to use the SMO inhibitors sonidegib or vismodegib in SHH-driven medulloblastoma, preferably within clinical trials. Response to SMO inhibitors can be predicted by high-throughput genomic methods (30). It however remains unclear whether the SHH signaling pathway should be investigated for downstream mutations before initiating SMO inhibition, as adults tend to have mutations at the level of SMO or above, which do not confer primary resistance to these targeted agents (30). Patients with an initial response to SMO inhibition typically relapse in the subsequent course (31). Resistance to SMO inhibitors may be overcome by novel agents (32). As the spectrum of druggable targets may change in relapse (33), recurrent tumors should be re-biopsied before initiating SMO inhibition.

Systemic treatments such as MEMMAT (metronomic and angiogenesis inhibition, NCT01356290) (34), TOTEM (topotecan and temozolomide, NCT00918320) (35), or TEMIRI (temozolomide, irinotecan) (36) [some of these approaches supplemented with bevacizumab (24, 37) and/or temozolomide (35–37)], are not specific for particular subpopulations, although their rationale is also found in a systems-biology and therefore pathophysiology-based approach.

The increasing complexity of medulloblastoma classification (38, 39) makes it likely that novel druggable targets will soon be available. For example, among the newly described non-WNT- and non-SHH-driven medulloblastoma subgroups (39), ~40% of patients harboring *MYC* amplifications may be candidates for immune checkpoint inhibition. It is probable that patients will be clustered in increasingly small subgroups in the future, thus generating a high demand for high-throughput diagnostic methods and also raising the question of financial reimbursement within the healthcare systems. Other recent clinical trials enroll small, highly selected populations, i.e., for immune checkpoint inhibition studies (NCT03838042, NCT03130959).

Liquid biopsies of CSF, serum, and urine that contain tumor cells, circulating cell-free DNA, extracellular vesicles, or proteins are currently being investigated and may provide a less invasive tool for early detection of tumor load, recurrence, and druggable targets (40), as demonstrated for other entities (41).

## Pineal Region Tumors (PRTs)

Incidental diagnosis of pineal region masses occurs in about 1–10% while diagnosis during autopsy of pineal masses occurs in 20–40% of cases (42). Nonetheless, the majority of these masses are benign and asymptomatic requiring observation as only approach. Pineal tumors are rare, representing 0.5 to 1% of adult CNS tumors in Europe and the United States, and 4% in Japan. They belong to five different histological entities: (i) tumors of the pineal parenchyma (representing 25–30% of all cases), (ii) germ cell tumors -GCT-, (iii) glial tumors and, (iv) metastatic location of systemic cancer and (v) papillary tumor of the pineal region.

PRTs most often manifest initially as non-specific neurological signs related to tumor site. Symptoms related to their growth are: hydrocephalus (due to aqueduct of Sylvius obstruction), Parinaud syndrome (compression of superior colliculi) or endocrine disturbances. Once that pineal origin of tumor is confirmed an adequate staging through review of CNS imaging should be done in parallel with a complete physical and clinical examination. GCT is the most probably diagnosis if a second location is observed in supra-optic region while detection of second location elsewhere suggest lymphoma or metastasis as alternative diagnosis. Some malignancies like atypical-teratoid/rhabdoid tumor (AT/RT), CNS embryonal tumors, pinealoblastoma, or lymphoma should be suspected when there is a meningeal enhancement.

To achieve a complete staging a spinal MRI (if possible before surgery), CSF cytology (during surgical shunting or by lumbar puncture) should be performed. Evaluation of markers (AFP, total and free HCG chain) in serum and CSF is also important to avoid unnecessary primary surgery.

Ophthalmologic examination will focus on visual acuity and field and rule out a lymphomatous pseudouveitis diagnosis. Endocrinological investigations are needed to rule out diabetes insipidus, or a sexual and/or growth hormones deficiency even if no supraoptic mass is detectable. When hydrocephalus is present, urgent shunting should be discussed.

Then, a multidisciplinary medical neuro-oncology board should discuss the necessity and the technical tools to obtain pathological proof if required, according to age, race and lesion staging. PRT may occur at any age.

Among adolescent and young adults, CGT represents the most common diagnosis. However, gliomas and tumor of the pineal gland including pinealocytoma or pinealoblastoma should be suspected. Gliomas, lymphomas, and metastases are more frequent in adults. Pineal cysts should be discarded whatever the age.

GCT are more frequent in Asians (43, 44). Chemotherapy as primary approach should be performed in patients with positive markers (blood and/or CSF), lymphomatous cells in CSF and/or specific alterations of slit lamp. Nonetheless, pathological specimens and diagnosis should be obtained through surgical resection or biopsy performed as open biopsy or during ventriculocisternotomy. Neurosurgeons expert in this region of the brain should perform surgery.

The treatment will then be tailored according to each pathology. The WHO 2016 classification proposes a three-stage classification (grade I, II or III, and IV) of tumors of the pineal parenchyma. The MIB1 proliferation index is increasing between grade I to IV (45). DNA methylation profiling of tumors of the pineal region confirms these three distinct subgroups: pinealocytoma, pinealoblastomas and pineal parenchymal tumor of intermediate differentiation (PPTID) (46).

### Pinealocytoma

Pinealocytoma is the most differentiated tumor type. It shows no mitosis or necrosis. Neuronal and/or neuroendocrine markers are present (neurofilaments, synaptophysin, chromogranin A) with sometimes S antigen and rhodopsin resembling the pineal phylogenesis. It lacks genomic copy number abnormalities. It occurs mostly in adults above 40 years old. Treatment includes complete surgery when feasible, with no further treatment.

### Pinealoblastoma

Pinealoblastoma is the most aggressive tumor (grade IV), and is defined as an embryonal undifferentiated tumor, with monomorphous small blue round cells that mimic those of any other CNS embryonal tumors, particularly medulloblastoma. It may show neuroblastoma-like Homer-Wright pseudorosettes or retinoblastoma-like Flexner Wintersteiner rosettes. It expresses INI1 that allows ruling out AT/RT. DNA copy number profiling suggests a close relationship between CNS-embryonal tumors and pinealoblastoma (47). Recurrent variants are found in genes involved in PKA- and NF-κB signaling pathways, and chromatin remodeling machinery (48). Mutually exclusive DICER1 mutation, DROSHA homozygous deletion and absence of KBTBD4 distinguish pinealoblastoma (49). Two peaks of incidence could be observed in pinealoblastoma incidence: the first in the two first decades of life and the second before 10 years old. Methylation profiles differ between pediatric and adult pinealoblastoma suggesting that pediatric tumors arise *de novo* while adult pinealoblastoma may arise from a pineal parenchymal tumor or a normal pineal gland. DNA methylation profiling reveals three subtypes and two new subgroups that are exclusively present before 7 year of age: (i) Pinealoblastoma-RB displays similarities with retinoblastoma and, (ii) Pinealoblastoma-MYC when MYC activation is present. Alterations within the miRNA processing pathway (affecting DROSHA, DGCR8, or DICER1) are found in about two thirds of cases in the three core subtypes (46). Treatment strategies mimic those for other malignant embryonal brain tumors. Significant treatment experience has been gained in pediatrics (50). After maximal resection, an application of chemotherapy (often platinum based) with radiation (usually CSI with local boost) is proposed. Using this strategy in 10 adults, a 3-year PFS of 68% was reported (51). Out of 45 patients aged 18 years or above, with malignant pineal tumors treated with radiotherapy followed by chemotherapy (in 34/45 patients), the median OS was 100 months. The extent of initial disease, differentiation (PPTID vs. pinealoblastoma), and residual disease significantly influenced OS as independent factors in a multivariate analysis. Local control was better in patients older than 32 years. Spinal control was increased in PPTID. Treatment failures occurred more than 5 years after treatment in 20% of cases. The median survival after relapse was 15 months. (51). Out of 135 patients with pinealoblastoma aged 0.01 to 20.7 years (median 4.9), favorable prognostic factors for progression free survival (PFS) were age, administration of radiotherapy and M0 staging (52).

### Pineal Tumor of Intermediate Differentiation (PPTID)

PPTIDs are tumors that fall between pinealocytoma and pinealoblastoma. They are composed of round cells with abundant cytoplasm and “poivre et sel” chromatin. Some necrosis or endothelial proliferation may exist. There are no consensual grading criteria between grade II and III according to WHO classification. Pinealoblastomas and PPTIDs share similar structural and numerical abnormalities including alterations of chromosome 1 and loss of chromosomes 20 and 22. The *KBTBD4* small in-frame insertion as well as the absence of *DROSHA* deletion or *DICER1* mutation characterized PPTID (49). They occur in people between 20 and 40 years. Due to their rarity management of these tumors still remains not clearly defined. Surgery with complete removal is the best treatment option. Local or CSI is usually recommended in grade III tumors.

Other pineal tumor types may also be diagnosed, such as GCT (discussed in further chapter), glial tumors and metastases from other tumor types.

### Glial Tumors of the Pineal Region

Glial tumors include pilocytic astrocytomas, high-grade gliomas, ependymomas and papillary tumors of the pineal gland (PTPG).

Low-grade gliomas may be also observed. They may be difficult to diagnose on tiny biopsies and may be confused with piloïd gliosis of a pineal cyst or of gliosis surrounding another tumor type. The presence of a KIAA1549:BRAF fusion transcript or its variants is of major diagnostic value. Gangliogliomas were also reported in this location (53). The treatment does not differ from that of low-grade gliomas located elsewhere. Glioblastomas are very rare. They display features of diffuse midline and non-midline gliomas. Median age at diagnosis is 50 years. Only one case showed the histone H3 K27M-mutation while no evidence of IDH-1 R132H mutation or 1p/19q co-deletion are available. Nonsense mutations in *ATRX* and *NF1* could be showed by targeting exome sequencing. Despite adjuvant radiation and chemotherapy, median OS is 15 months (54). Case reports of pineal gliosarcoma have been published (55).

### Papillary Tumors of the Pineal Region (PTPR)

PTPRs are very rare neuroepithelial tumors issued from the subcommissural organ. They are characterized by an epithelial-like, loose papillary growth pattern, expression cytokeratins, and less often EMA and GFAP. Genome wide profiling identify loss of chromosome 10 in all and other chromosome imbalances. *PTEN* mutations are seen, with activation of the PI3K/Akt/mTOR signaling pathway (56). PTPRs overexpress SPDEF, a rodent subcommissural organ gene. DNA methylation profiling reliably distinguished PTPR from ependymomas and PPTID. Two groups with clinical relevance were proposed (57). They affect children and adults (mean age at diagnosis 35 years). A recent cohort of the 177 cases published so far, showed that 56% recurred after a median 29 months with a 36-months survival rate of 83%. After adjustment for age, tumor size and surgical treatment were associated with survival. However, no significant benefits from GTR or adjuvant treatments including radiotherapy were reported (58).

## Glioneuronal Tumors and Rare Glial Tumors

### Pilocytic Astrocytoma

Pilocytic astrocytomas (PAs) represent 5.4% of gliomas and are most common during the first two decades of life and in young adults. They are the principal CNS neoplasms associated with neurofibromatosis type 1, particularly those involving the optic pathways. In adults, the onset is earlier as compared to diffuse astrocytomas. The definition by WHO 2016 is that of an astrocytoma with a biphasic pattern composed of a variable percentage of compacted bipolar cells with Rosenthal fibers and loose, textured multipolar cells with microcysts and occasional granular bodies. The tumor is WHO grade I, but cases with anaplasia may occur. IDH 1-2 mutations are absent, while specific fusions are present fusing BRAF with KIAA1549 or other partners.

Preferred locations are optic nerve/chiasm, hypothalamus/thalamus, cerebellum, brain stem, cervico-medullary junction and cerebral hemispheres with focal neurological deficit, endocrinopathy and raised intracranial pressure as the most frequent symptoms at onset. On MRI, PAs classically appear as cystic masses with an enhancing mural nodule.

The standard of care consists of a GTR for accessible lesions (i.e., cerebellum, cerebral hemispheres) with a 10-year survival rate of 100% as compared to 74% for subtotal resection (59). Resection is more problematic in critical locations, such as optic pathways, thalamus, and hypothalamus. Exophytic portions of tumors in brainstem and cervico-medullary junction are resectable. Adjuvant radiotherapy for incomplete resection/biopsy prolongs PFS but equivalent OS are reported with observation and salvage radiotherapy. IMRT and proton facilities may be useful to minimize damage to surrounding normal structures. Chemotherapy is used to avoid/delay radiotherapy in children or as salvage treatment (carboplatin + vincristine, temozolomide, multidrug combinations) in adults. BRAF inhibitors have been proposed for patients with BRAF V600E mutation.

### Pleomorphic Xanthoastrocytomas

Pleomorphic xanthoastrocytomas (PXAs) represent <1% of astrocytic neoplasms, sometimes associated with neurofibromatosis type 1. The definition of WHO 2016 is that of an astrocytic glioma with large pleomorphic and frequently multinucleated cells, spindle and lipidized cells, a dense pericellular reticulin network, numerous eosinophilic granular bodies, and often a neuronal differentiation. It is a grade II tumor, but in up to 30% of cases, anaplastic features (WHO grade III) are present. IDH 1-2 mutations are absent; MGMT promoter methylation is rare, while BRAF V600E mutation is observed in 50–70% of cases. MRI shows a supratentorial lesion, more often in the temporal lobe, involving the cortex and overlying leptomeninges, frequently cystic, with moderate or strong contrast enhancement.

As for management (60), GTR, when feasible, is the standard of care but frequently a local progression occurs. Eighty percent of patients undergo reoperation alone or followed by radiotherapy, while 20% of patients receive radiotherapy alone. In 30% of patients, chemotherapy (temozolomide, bevacizumab, CCNU, lapatinib), is also used. There are increasing reports of the efficacy of BRAF inhibitors (vemurafenib, dabrafenib), alone or associated with MEK inhibitors (cobimetinib, trametinib) (61).

### Subependymal Giant Cell Astrocytomas

Subepedymal giant cell astrocytomas (SEGAs) occur in patients with tuberous sclerosis complex (TSC). The TSC is an autosomal—dominant genetic disease characterized by gene mutation of hamartin (TSC1) or tuberin (TSC2). These proteins are generally involved in inhibition and suppression of mTOR pathway, which is known to be involved in cell proliferation. Children are mostly affected.

SEGAs are WHO grade I lesions localized most commonly in the region of the foramen of Monro. These lesions arise from subependymal nodules covering ventricles.

The most common symptoms are pharmacoresistant seizures, while MRI shows a homogeneous enhancing lesion.

Surgery represents the best treatment option for lesions associated to increasing growth or symptomatic lesion presenting for example with obstructive hydrocephalus.

Due to the location, morbidity following surgical resection is observed in 20–50% of patients. In the last 10 years everolimus, an m-TOR inhibitor, is being increasingly used also as first-line treatment, due to a clear activity on tumors and seizures. In a phase III trial patients were randomized to receive everolimus or placebo. Of note, patients receiving everolimus had a radiographic response rate of 35% compared to 0% in patients receiving placebo (62). In addition, seizures are reduced in up to 70% of patients. Responses to everolimus last for several years, and overall, the outcome of SEGAs is favorable but the patients are mentally retarded and suffer from seizures.

### Gangliogliomas

Gangliogliomas (GGs) are rare tumors accounting for 0.5–1.3% of all primary CNS tumors and are more frequent in children than in young adults. They are composed of dysplastic ganglion cells in association with neoplastic glial cells. Neuronal component is recognized by antibodies to neuronal proteins (neurofilaments, synaptophysin, chromogranin A), while the glial component is immunostained for glial fibrillary acid protein (GFAP). IDH 1/2 mutations and 1p/19q codeletion are absent, H3K27M mutation can be found in midline tumors, while BRAF V600E mutation is observed in 20–60% of cases (63). BRAF V600E mutation is associated with mTOR pathway activation in dysplastic neurons. The prognostic role of molecular alterations is unclear. Anaplastic GGs correspond histologically to WHO grade III and the frequency varies from 1 to 6%.

On MRI, lesions are cortically-based and well-circumscribed, either solid or cystic with a mural nodule, and calcifications are present in ~50% of patients. Contrast enhancement may be absent or present in variable degree.

Surgical resection is the main therapeutic option, and GTR is often feasible in the hemispheric locations, and is the best predictor of prolonged PFS and OS. Seizure freedom following GTR is obtained in 70–90% of patients. Early surgical resection, shorter duration of epilepsy (≤1 year), younger age and absence of secondary generalized seizures are associated with better seizure control (64). Grade I GG patients have an OS of 84–94% at 10 and 15 years. Malignant transformation of GGs varies from 2 to 5%. Conformal radiotherapy can be used as adjuvant treatment in incompletely resected GGs but it is unclear whether it is better than surveillance postponing RT at progression. Rarely, recurrent tumors respond to nitrosoureas, temozolomide, etoposide, or platinum compounds. Responses to BRAF-mutated tumors to BRAF inhibitors, alone or with MEK inhibitors, have been reported suggesting that these drugs can be an important treatment option (61).

## Ependymal Tumors

Ependymomas constitute 8–10% of brain tumors in children and 1–3% of primary brain tumors in adults (65). The small number of patients included in the studies, the heterogeneity in terms of age and location as well as the long time period analyzed explain the lack of evidence-based treatment strategies. Overall, adults have a better prognosis than children with 5-year survival rates of 56–85%compared to 36–64%, respectively.

In adults, ependymomas definitely prevail in the spinal cord (the cervical and thoracic locations are more common), while in children they prevail in the posterior fossa (60%), and are typically located in the fourth ventricle or in the cerebello-pontine angle. Ependymomas in the supratentorial compartment are more frequent in adults (50–60%) as compared to children (30%), and are located in the lateral or 3rd ventricles (60%) or in the brain parenchyma (40%). They are more often high-grade tumors.

According to WHO 2016, diagnosis of anaplastic ependymoma can be done in ependymal tumors with associated high mitotic rate, high cell density, necrosis, and widespread microvascular proliferation. Histopathological grading has been for many years a matter of discussion; however, as several recent studies (66) have demonstrated a different outcome for ependymomas grade II and grade III, this distinction is still used for clinical decision.

Recently, molecular profiling has identified nine subtypes of ependymomas (the subtypes in each location) with different outcomes (67). In particular, the variant RELA fusion (supratentorial location and poor outcome) has been added in the updated WHO 2016 classification.

### Intracranial Ependymomas

About pattern of failure, incidence of CSF dissemination at time of diagnosis is <5% but increase to 15% in patients with anaplastic and infratentorial tumors. It is also important to observe that majority of tumor recurrences (up to 90–95%) happens due to the lack of local tumor control, while the occurrence of extraneural metastases is extremely rare (<1%).

After surgery, a craniospinal MRI and an assessment of CSF cytology are mandatory (not earlier than 2 weeks after surgery, to avoid confusion with peri-operative tumor spill). Regular surveillance with MRI of the entire CNS could discover asymptomatic recurrences (43%) and impact subsequent treatment and survival (68). Open questions are how often and for how long the surveillance should be performed and when CSF cytology is needed.

Surgery is the mainstay in the management of ependymomas (69). Gross total resection (GTR) is the most important prognostic factor for patients with localized disease, and image-verified (MRI) gross total resection is achieved in 50–75% of patients. GTR is dependent upon tumor location and infiltration. The definition of GTR is crucial in order to define risk classification and guide treatment decisions. Second-look surgery following initial incomplete surgery is increasingly advocated (especially in children), assuming that a complete resection is achievable. GTR cannot be performed at primary surgery due to:

(1) patients in extremis immediately prior to surgery; (2) findings during operation different from predicted at imaging; (3) neurosurgeon experience.

Open issues are when the second surgery is to be performed (as soon as possible? After a period of observation?). A second surgery is recommended whenever feasible at recurrence.

Conformal radiotherapy is part of the standard of care as adjuvant treatment for patients with anaplastic (grade III) ependymoma and for patients with grade II ependymoma after incomplete resection. Doses of 54–55 Gy for grade II tumors and 60 Gy for grade III tumors are commonly used. There is no evidence of efficacy for radiotherapy after GTR of a grade II tumor (70).

CSI was the SCI for many years, based on the propensity of ependymomas to spread via the CSF, but recent studies report no differences in outcome (including the rate of spinal metastases) when larger treatment volumes have been used. Nowadays, limited field radiotherapy is used for localized tumors (MO) and CSI is restricted for patients with metastatic disease (M1) (69). Reirradiation at recurrence can be performed using a full course either of fractionated irradiation or stereotactic irradiation or, in some specialized centers with proton therapy.

Durable responses following reirradiations can be achieved. In addition, durable survival has been observed with resections of localized metastases, being salvage surgery another option. Chemotherapy, especially in adults, is used especially at recurrence with drugs such as platinum compounds (71) or temozolomide (72).

### Spinal Cord Ependymomas

The two histologic subtypes are represented by myxopapillary ependymoma (grade I) arising in the cauda equina and classic ependymoma (grade II or III) arising more often in the cervical spinal cord (65, 73).

The risk of CSF spread in classic ependymoma is rare. Grade I myxopapillary ependymoma could have a worse prognosis than grade II tumors, due to a lower frequency of total resections and increased risk of mechanical CSF disseminations. Re-operation should be performed whenever feasible.

Post-operative conformal radiotherapy prolongs PFS in incompletely resected tumors. A risk of radiation-induced myelopathy should be considered. Reirradiation, also with stereotactic techniques, can be useful for local recurrence. Chronic oral etoposide for patients relapsing despite surgery and radiotherapy has been reported with some efficacy.

## Grade III Meningioma and Mesenchymal Non Meningothelial Intracranial Tumors

### Grade III Meningiomas

Anaplastic meningiomas account for ~1–3% of meningiomas and exhibit malignant cytology (resembling that of carcinoma, melanoma, or high-grade sarcoma) and/or markedly elevated mitotic activity (1). In addition to high mitotic activity many anaplastic meningiomas show extensive necrosis and Ki-67 tumor cell proliferation indices of 20% or higher. According to the WHO classification of tumors of the CNS, anaplastic meningiomas correspond to grade III. The rare histological rhabdoid and papillary meningioma variants also correspond to grade III. They have high recurrence rates and may metastasize to extracranial sites. The prognosis of anaplastic meningiomas is poor with median OS below 1 year.

Molecularly, anaplastic meningiomas show high rates of NF2 aberrations. Hotspot mutations in the TERT promoter were found in 20% of WHO grade III meningiomas (1.7 and 5.7% in WHO grade I and II meningiomas, respectively) and are associated with unfavorable outcome (74). In rhabdoid meningiomas, germline, and somatic mutations of BAP1 gene have been identified and may be linked to unfavorable outcomes (75). Epigenetic signatures have been shown to correlate with clinico-pathological features in meningiomas and anaplastic meningiomas were associated with the unfavorable specific methylation profiles (MC-mal and MC-int B) (76).

Therapeutic strategy for anaplastic meningiomas at initial diagnosis should aim at radical resection followed by fractionated radiotherapy at a dose of at least 54 to 60 Gy delivered in 1.8–2.0 Gy fractions (potentially with a 10 Gy boost on the remaining tumor volume after subtotal resection) (77). No evidence-based treatment recommendations for recurrent anaplastic meningioma exist, but usually resection or radiotherapy is recommended, if possible. Pharmacotherapy of anaplastic meningiomas remains experimental and no efficacy for specific drugs has been proven.

### Solitary Fibrous Tumor/Haemangiopericytoma

Incidence of the Solitary fibrous tumor/haemangiopericytoma is <1% of all primary CNS tumors. It occurs most commonly in adults and is typically dura-based. Histologically, solitary fibrous tumor/haemangiopericytoma appears as a mesenchymal tumor with fibroblastic pattern. Genomic inversion at the 4 12q13 locus resulting in the *NAB2* and *STAT6* genes fusion can be frequently observed. Detection of nuclear STAT6 expression or detection of *NAB2-STAT6* fusion is highly recommended to confirm the diagnosis of solitary fibrous tumor/haemangiopericytoma (78–81). Two main phenotypes are described: the classic solitary fibrous tumor phenotype is characterized by pattern-less histomorphology, rich collagen matrix and scarce mitoses. In contrast, the haemangiopericytoma phenotype shows high cellularity and increases mitotic activity and necrosis (1). These two phenotypes belong to a same tumor spectrum of intermediate malignancy with a risk of local relapse and metastasis increasing with higher mitotic activity and necrosis. Drug treatment of solitary fibrous tumor/haemangiopericytoma is experimental, as no effective agents have been identified. The antiangiogenic multi tyrosine kinase inhibitor axitinib showed some promising activity in a small uncontrolled phase II study enrolling advanced or progressive solitary fibrous tumors and should be validated in further studies (81, 82).

## Primary CNS Lymphoma

Primary cerebral lymphoma (PCL) is a rare form of non-Hodgkin lymphoma representing <5% of primary cerebral tumors (83). It may be associated with lymphomatous locations in the meninges (15–20%), the eye (10–20%) and the spinal cord (<5%) but is defined by the absence of any systemic lesion. Immunodeficiency, mainly in the setting of AIDS or organ transplant, is the only well-recognized risk factor, but since the improvement of antiretroviral therapy in AIDS, it mainly arises in immunocompetent patients, with a median age of 65 to 70 years (84).

The clinical presentation is usually sub-acute and non-specific, depending on the location of the disease in the brain. On MRI, the disease typically presents as one or several deep and periventricular expansive lesion(s) with typical intense and usually rather homogeneous contrast enhancement after gadolinium infusion (85), but there can be atypical presentations, mimicking other brain tumors, or without contrast enhancement. In immunocompromised patients, contrast enhancement is most frequently peripheral with necrosis in the center (86). Perfusion, diffusion and spectroscopy sequences can be useful to direct toward the diagnosis (87).

In most cases, the diagnosis is confirmed by a brain biopsy that should be performed before the introduction of steroids which can cause false-negative biopsies (vanishing lymphoma). In the case of an associated meningeal or ocular involvement, the brain biopsy can be avoided if the aspect of the lesion is typical on MRI and if monoclonal lymphomatous cells are found on CSF or vitreous analysis. All suspected patients should have a lumbar puncture with cytological and flow cytometry analyses and an ophthalmological examination with at least slit-lamp and fundus examination before considering a brain biopsy. In more than 90% of cases, the pathological diagnosis is a diffuse large B-cell lymphoma, mostly of ABC type (88). The tumor is usually EBV positive in immunocompromised patients and EBV negative in immunocompetent patients. The staging should also include a full body CT scan and/or FDG-PET scan to exclude a systemic location of the disease (89).

Treatments usually used in systemic lymphomas are not efficient in PCL. The treatment of PCL consists of an intravenous high-dose methotrexate (HD MTX) based polychemotherapy. The use of rituximab is controversial (90). The tumor is highly chemosensitive with 40–60% of complete response to chemotherapy (91–94), but relapses are very frequent, notably within the first months after the end of the treatment. In order to reduce the risk of relapse, a consolidation treatment can be delivered to patients with good response to initial chemotherapy, with two options: whole-brain radiotherapy (WBRT) or high-dose chemotherapy with autologous stem cells transplantation (HCT-ASCT) (93–95). However, HD MTX combined with whole-brain radiation therapy (WBRT) exposes patients to a risk of delayed neurotoxicity (96). This risk is particularly important in elderly patients who frequently develop dementia and severe gait disorders after treatment so that WBRT has been gradually abandoned in patients older than 60 years. HCT-ASCT is restricted for patients under 60–65 years in good clinical condition.

Age and Karnofsky performance status are the most well-recognized prognostic factors, while the prognostic value of other factors is controversial (CSF protein, blood LDH, early response to chemotherapy or molecular factors such as chromosome 6q loss for instance) (97, 98). The prognosis for the disease remains severe, with a median OS of 30–60 months in most studies, but the percentage of long-term survival has increased (5 year-OS of 38%) as showed in a recent population-based study (99).

## Germ Cell Tumors

Intracranial germ-cell tumors (GCT) derive from the primordial germ cell: their aberrant migration occurs axially (i.e., suprasellar, pineal regions with 10% being bifocal).

Pathology may include one (pure) or several (mixed) of the following components that may not all appear in a limited biopsy. Germinomas, the most frequent histology, are composed of undifferentiated cells with some syncytiotrophoblastic cells (explaining why germinomas may secrete ß-human chorionic gonadotropin (HCG) intermixed with an inflammatory infiltrate. Immunohistochemistry stains positive for placenta-like alkaline phosphatase (PLAP). The other components may show aberrant differentiation toward extraembryonic and/or somatic features: yolk sac tumors stain for alpha fetoprotein (AFP) and glypican 3; choriocarcinomas are composed of mononucleated (cytotrophoblastic) and multinucleated (syncytiotrophoblastic) cells that stain for HCG; and embryonal carcinomas stain for CD30. Teratomas are either mature, with the presence of all three germ layers, or immature, with varying components of immature mostly neural tissue. Each component has its specific sensitivity to treatment, germinomas being exquisitely sensitive, teratomas resistant to radio-chemotherapy (indicating the need for surgical removal), and other components being somewhere in-between.

There is a male predominance, especially for germinomas, a racial predominance (Asiatic) and an age distribution: teratomas and pure yolk sac tumors are mainly restricted to infants and children whereas all others have a peak incidence during adolescence and early adulthood and show isochromosome 12p or 12p amplification.

In the pineal location, Parinaud's syndrome and/or raised intracranial pressure is recognized promptly. Suprasellar location symptoms—diabetes insipidus, impairment of visual fields, or variation in onset of puberty—are often overlooked for long periods in adolescents. Basal ganglia germinomas occur in Asiatic patients, as a longstanding history of focal deficit and/or mental deterioration.

Pineal tumors may extend to the third and/or fourth ventricle and invade the thalamic region. Suprasellar masses involve the pituitary stalk and third ventricle and may compress or invade the optic chiasm. Primary tumors tend to propagate along the ventricular walls often causing endocrinological disorders even when they are not radiologically detectable: disappearance of the posterior pituitary bright spot correlates to diabetes insipidus. Germinomas are iso- to hyperdense, sometimes calcified on computer tomography scan, iso- or hypointense on T1- and T2-weighted MRI, with moderate to marked homogeneous gadolinium enhancement, a drop in apparent diffusion coefficient values. The diffusion-weighted imaging mimics that of Langerhans cell histiocytosis and lymphocytic hypophysitis, but differs from craniopharyngiomas and gliomas. Bifocal tumors (if pineal and suprasellar) are a specific entity, not a metastatic disease and are typical of GCT. Teratomas may be suggested when the three compartments (parenchymal, fat, and calcifications) are present. Basal ganglia germinoma MRI shows atrophy of the cerebral peduncle, basal ganglia and/or cerebral hemisphere. Spinal MRI, ideally performed preoperatively to avoid bloody artifacts, should look for nodules and/or linear enhancement. If doubtful, it should be repeated with both sagittal and axial sequences.

Markers should be measured both in serum and CSF. Total HCG is secreted by choriocarcinomas and at lower levels by germinomas and embryonal carcinomas. Total HCG measures both HCG and subunit β and is expressed as mUI/ml. Positive cytology of CSF is found in 10% of cases.

The initial strategy requires a multidisciplinary approach. The initial workflow is crucial and includes craniospinal MRI, determination of markers (AFP and HCG) both in blood and CSF, cytology of CSF. The incidence of relapses increased from 4 to 24% if incompletely staged germinomas. Apart from the Japanese strategy (explained later), if markers (serum and/or CSF) are positive, or for non-secreting bifocal tumors (most likely germinomas) histological confirmation is not required. Otherwise, open surgery, stereotactic biopsy, or endoscopic third ventriculostomy may be used to obtain histological proof. The latter is often used to relieve hydrocephalus and allows inspection of the ventricles. Detection of ventricular nodules during ventriculoscopy does not qualify the patient as metastatic, if they are not detectable on MRI.

American and European groups define two-categories: germinomas and non-germinomas. Localized germinomas were initially treated within the SIOP (International Society of Pediatric Oncology) GCT 96 protocol with either CSI of 24 Gy with a local boost of 16 Gy, or combined treatment with etoposide–carboplatin–ifosfamide (CarboPEI) followed by a 40-Gy focal boost (100). Due to potential neurotoxicity in the former regimen, and excess of ventricular relapses in the latter, the current CGTII SIOP protocol explores the same induction chemotherapy followed by whole ventricular irradiation (WVI) of 24 Gy with a 16-Gy boost, delivered exclusively in patients who do not reach complete remission (CR) prior to radiation. The Children's Oncology Group (COG) launched ACNS 1123 that explores combined treatment by etoposide–carboplatin, followed by 18 Gy of WVI with a 12-Gy boost in patients reaching CR, or 24 Gy and 12 Gy respectively otherwise (101).

Localized non-germinomas were treated in the two successive SIOP trials with ifosfamide–etoposide–cisplatin (PEI) followed by a 54-Gy boost to the primary. As SIOP-GCT96 cohort reported a poorer prognosis for patients with AFP>1,000 and/or with persisting viable tumor after chemotherapy (102), SIOP-GCTII explores the role of high-dose therapy followed by stem-cell rescue, prior to radiation, in these high-risk patients. ACNS 0122 delivered etoposide–carboplatin–ifosfamide with 36 Gy of CSI and a 18-Gy boost for patients in CR, and consolidation by high-dose etoposide thiotepa with stem-cell rescue followed by CSI otherwise. Five-year OS was 93%. The current ACNS 1,123 for non-germinomas explores the feasibility of reducing the field to WVI. The overall prognosis for patients with recurrent germinomas remains excellent. Non-germinomatous GCT that recur may be salvaged, although the long-term outcome is worse.

As all Japanese patients benefit from histological documentation, a three-category strategy is used (103). Pure germinomas reached 2-year OS of 97% when treated with three courses of etoposide and carboplatin concomitant with WVI of 24 Gy. The intermediate group (HCG-secreting germinomas and mixed tumors composed of germinomas and immature teratomas) received similar treatment, with an increased dose to the ventricles (30 Gy) and a local boost (20 Gy); their 10-year OS was 89%. The high-risk group (non germinomatous tumors) received three courses of platinum–etoposide–ifosfamide concomitant with whole-brain irradiation (30 Gy) with a local boost (30 Gy) and spinal (24 Gy) radiotherapy; their 10-year OS was 58%. In the ongoing study, strategy for the intermediate group includes similar chemotherapy with a decreased dose to the ventricles (23.4 Gy) and an increased boost to the tumor bed (27 Gy). The high-risk group receive a 30.6 Gy CSI plus 30.6 Gy additional boost to the tumor with concomitant and maintenance courses of platinum–etoposide–ifosfamide.

Biology of intracranial (non-)germinomas is lagging behind due to the lack of availability of tumor tissue in most chemo-naive patients. Intracranial and extracranial tumors have similar mutations in KIT (26%), KRAS (15%), NRAS (5%; exclusive from KIT), mTor (8%), CBL (11%), with copy gain of AKT1 (19%). Potentially effective drugs in the future include sunitinib and mTOR inhibitors. Embryonal carcinoma is CD30-positive and could benefit from brentuximab vedotin.

Liquid biopsy, especially of the CSF, is an attractive surrogate for tissue sampling; miRNAs are potential candidates for the follow-up of tumor-mass response to therapy (104).

## Spinal Cord Tumors

Primary tumors of the spinal cord are uncommon neoplasms representing 2–4% of all primary tumors of the CNS in adults (105). The overall incidence of malignant and non-malignant tumors was 0.97 per 100,000, with only 30% of all spinal cord tumors being malignant. These tumors can be divided into extradural and intradural lesions. Extradural tumors account for almost 60% of spinal cord neoplasms and in most cases are primarily metastatic in origin; in particular, they derive from lung, breast, prostate, and lymphoma. Moreover, osteosarcoma, chondrosarcoma, or multiple myeloma can arise from the vertebral column.

Intradural tumors can originate in the parenchyma of the cord (intramedullary lesions) or in the thecal sac but external to the spinal cord (extramedullary lesions). Symptoms related to spinal tumors can depend on tumor location and size, causing back pain, numbness, temperature sensation disturbance, radiculopathy and other sensorimotor deficits. Magnetic resonance imaging is the gold standard to assess these lesions and CT scans can be useful for imaging the osseous spine and when the primary lesion infiltrates the vertebra.

### Intradural, Extramedullary Tumors

Extramedullary tumors account for more than 70% of intradural spinal cord neoplasms in adult patients. Most frequent extramedullary spinal cord tumors include meningiomas (discussed previously), nerve sheath tumors (schwannomas and neurofibromas).

### Nerve-Sheath Tumors

Nerve-sheath tumors account for 27% of the primitive spinal cord neoplasms. Almost all are non-malignant neoplasms (98%) with a male-female rate ratio of 1.11 (105).

Schwannomas are slow-growing lesions that arise from the dorsal nerve root in most cases. The average age of onset is 40–60 years and these lesions are almost never malignant. Usually, schwannomas present as solitary tumors and multiple spinal schwannomas are often encountered in patients with neurofibromatosis type 2 (NF2) (106). Surgery is recommended in case of symptomatic or growing tumors and GTR can be reached in most cases. Radiotherapy should be used with caution and only when absolutely necessary, especially in hereditary diseases; indeed, in these patients the use of radiation therapy could increase the risk of a malignant tumor in the radiation field (107) Bevacizumab, an antiangiogenic drug, could also be beneficial in spinal cord lesions due to its promising activity in neurofibromatosis type 2-associated vestibular schwannomas (108); moreover, lapatinib and nilotinib in combination with radiotherapy demonstrated some benefit in a preclinical model of NF2 associated peripheral schwannoma (109).

Neurofibromas have characteristics similar to schwannomas and are classified as general or plexiform histology. Multiple neurofibromas can be associated with neurofibromatosis type I (NF-1) and, a small rate of patients can develop malignant peripheral nerve sheath tumors (MPNST) with dismal clinical outcome; the cumulative lifetime risk of developing MPNST in patients with NF1 is about 8–13% (110). The cornerstone of MPNST treatment remains surgical resection, but is often not possible due to expected damage to adjacent structures. However, the important role of surgery was confirmed in a recent experience of the French Sarcoma Group analyzing 353 patients with MPNST (111). Although, in this retrospective study tumors were located in the trunk in only 37% of cases overall, the authors showed a statistically significant impact of radical surgery in prolonging disease free survival (DFS) and overall survival (OS). Radiation therapy could be administered to large, high-grade MPNST whereas, there is no consensus on the use of chemotherapy for MPNST (112). Recently, a prospective trial evaluating selumetinib, an oral selective inhibitor of MEK 1 and 2 showed an interesting activity against plexiform neurofibromas with a partial response of 71% and a disease control rate of 100% (113).

### Intradural, Intramedullary Tumors

In adult patients, the most frequent intramedullary tumors are gliomas. The incidence of spinal cord gliomas has been reported to be 0.12 per 100,000. Among gliomas, astrocytomas, and ependymomas (as discussed previously) account for 60–80% of all spinal gliomas and the incidence has been increasing over the last 30 years (114).

### Diffuse Gliomas

Astrocytomas account for 30–40% of all intramedullary spinal cord tumors in adults. The average age of onset is the third decade of life and they are predominantly located in the cervical region. About 90% of intramedullary astrocytomas are low-grade and glioblastomas rarely occur (about 1% of all astrocytomas). GTR is the gold standard treatment for pilocytic astrocytoma as these tumors are well-circumscribed. Diffuse and high-grade astrocytomas have an infiltrative pattern and GTR is rarely completed. In a systematic review, Hamilton et al. showed that GTR could improve OS both in low and high-grade gliomas while adjuvant radiotherapy only in high-grade lesions (114, 115). Chemotherapy could be used for relapse following radiotherapy. In a retrospective study, temozolomide showed interesting activity for recurrent low-grade spinal cord astrocytomas after surgery and radiotherapy: disease control rate was 73% and median progression free survival (PFS) was 14.5 months (116). In another retrospective study, bevacizumab reported partial response in 83% of cases and median OS of 22.8 months in recurrent high-grade astrocytomas (117).

Of note, spinal cord high-grade astrocytomas could be treated with targeted therapies; indeed, *BRAF* alteration status was demonstrated to be present in about 80% of low-grade spinal cord astrocytomas and these cases could be considered for a therapeutic approach with BRAF-MEK inhibitors. Moreover, the *H3F3A* K27M mutation, that could be a potential target of histone deacetylase or demethylase inhibitors, was reported in all cases of high-grade gliomas (118).

## Rare Pituitary Tumors

Important changes in the classification of non-neuroendocrine tumors have been performed in the 4th Edition of the WHO Classification of Pituitary Tumors (118). Non-neuroendocrine tumors are less frequent than pituitary adenomas, however it is important to achieve a differential diagnosis with other sellar masses (particularly those tumors arising in the posterior pituitary).

### Tumors of the Posterior Pituitary

Tumors arising in the posterior pituitary include the pituicytomas, the spindle cell oncocytomas, the rare sellar ependymomas and the granular cell tumors of the neurohypophysis (1, 118). According to the German Pituitary Tumor Register, pituicytomas account <0.1% of all sellar tumors (119). These tumors can be diagnosed as non-functioning pituitary adenomas due to their similar radiographic and clinical features.

About the clinical presentation of these tumors is mainly related to symptoms related to tumor size and compression. Thus, symptoms resulting from these tumors include: headaches, visual deficit, hyperprolactinemia, fatigue, and amenorrhea. These tumors had specific histological features. Nonetheless, Granular cell tumors and spindle cell oncocytomas shared the expression of thyroid transcription factor 1 (TTF-1). The immunohistochemical detected TTF-1 expression can be also observed in the specialized glia of the neurohypophysis, pituitary stalk and pituicytes. Moreover, tumor architecture and morphology is similar to normal pituicytes suggesting a shared pituicyte-lineage. All these data suggest that non-neuroendocrine pituitary tumors may originate from specific variants of pituicytes. Thus, it would be possible that granular cell tumors originate from granular variant, pituicytomas from the light/major variant and spindle cell oncocytomas from the oncocytic variant (118).

### Neuronal and Paraneuronal Tumors

Neuronal and neuroepithelial tumors may rarely arise in the pituitary and include gangliocytomas, neurocytomas, neuroblastomas, and paragangliomas. These tumor entities do not differ from those arising in other locations of the CNS. Briefly, we describe gangliocytoma and neurocytoma of the sellar region (1).

Gangliocytoma is a well-differentiated tumor, which is associated to a slow growth. These tumors had microscopic features similar to normal hypothalamic neurons (120, 121). When a glial component can be detected these tumors are called “gangliogliomas” (1). These tumors are generally associated to adenohypophysial pathology such as: acromegaly with impaired production of growth hormone-releasing hormone (GHRH), Cushing disease, precocious puberty and hyperprolactinemia (120, 121).

Microscopically, these tumors showed large mature ganglion cells with different size and shape. Abundant cytoplasm containing Nissl substance can also be found while cells can be binucleated or multinucleated. Patterns of stain include: synaptophysin, NeuN, MAP2, S100, and neurofilaments that highlight the axons and dendrites (122, 123). The glial elements can be identified with GFAP. Of note, Ki-67/MIB-1 is usually very low like mitotic rate.

Neurocytoma is a tumor composed of small neurons. Generally, this is a low-grade tumor with infrequent mitoses and Ki-67 proliferation index low than 2%. However, “atypical” neurocytoma can show higher mitotic rate (3 or more mitoses per 10 high power fields), Ki-67 expression (>3%), necrosis and microvascular proliferation. These tumors are associated to worse prognosis (1, 124, 125). Microscopically, these tumors are characterized by nuclei that range from round to oval with multiple nucleoli and finely granular chromatin. Cells are round with small—medium size converging in monotonous sheets and nests or rosette-like pattern within a vascular fibrillary neuropil. The cytoplasm is weakly eosinophilic. Occasional acidophilic hyaline globules within the neuropil resemble Herring bodies of the posterior pituitary. The immunohistochemical pattern reveals NeuN and TTF1 positivity in the nuclei and synaptophysin, chromogranin-A and neurofilaments in cytoplasm (126–128). Occasionally, S100 protein, calretinin and CD99 can be observed. Tumors associated to acromegaly and SIAD could also be express. Adenohypophyseal neuroendocrine tumors, olfactory neuroblastoma, and paraganglioma, represent differential diagnosis.

### Craniopharyngioma

Several cyst-like lesions may involve the pituitary gland and include both neoplastic and developmental lesions (1, 118). Clinically, they may present with signs and symptoms of a mass lesion impinging on the optic pathways and/or the hypothalamus. These cystic lesions and tumors include craniopharyngiomas, Rathke cleft cysts, epidermoid, and dermoid cysts, and arachnoid cysts (118). The 2016 WHO classification (1, 118) identifies two variants of craniopharyngioma, the adamantinomatous and papillary craniopharyngioma. It has been suggested that two different subtypes of tumors exists. This hypothesis has been suggested after the demonstration of distinct genetic mutations pathways of craniopharyngiomas. CTNNB1 gene mutations (encoding β-catenin protein) is exhibit by adamantinomatous craniopharyngiomas. This mutation results in aberrant nuclear accumulation of β-catenin, an immunohistochemically reliable tool for confirmation of the diagnosis. On the other hand, mutations of BRAF gene (BRAF V600E) are the main oncogenic alteration seen in papillary craniopharyngiomas; similarly, this mutation can be detected using BRAFV600E immunohistochemistry.

Recently several case reports showed dramatic tumor responses to BRAF-inhibitors alone or in association with MEK inhibitors.

### Germ Cell Tumors

The sellar/suprasellar region is the second most common site involved with germ cell tumors in the CNS with involvement of the hypothalamus, periventricular areas of the third ventricle, pituitary stalk and pituitary gland (118). Germ cell tumors of the CNS are histologically identical to the gonadal and extra-gonadal counterparts and may be divided pathologically into two major patterns: germinoma and non-germinomatous germ cell tumors. Germinomas are by far the most common involving this area. The differential diagnosis of germinomas in the sellar region includes inflammatory lesions and hematolymphoid tumors.

### Tumors of the Bone, Soft Tissues, and Peripheral Nerves

Lastly, tumors of the bone, soft tissues, and peripheral nerves may arise in the sella turcica area including chordoma and chondrosarcoma, meningiomas, solitary fibrous tumor/hemangiopericytoma, schwannomas, and other rare peripheral nerve sheath tumors. Briefly, we describe skull base chordoma.

### Chordomas of the Skull Base

Chordomas are malignancies of bone, which originate from ectopic residuals of the notochord. (129). These are rare tumors with an estimated incidence of 0.08 per 100,000 patients. Except for rare tumors originating from extranotochordal sites, the majority of these tumors take place in the sacrococcygeal region (50%), spheno-occipital region (35%) and vertebrae (15%) (129–131). These tumors arise more frequently in males with a peak of incidence in fifth and sixth decades (129). Chordomas are rare in adults below the age of 40 years old and exceptional in children and adolescents (<5%) (132, 133).

According to the WHO Classification of Soft Tissues and Bone chordomas can be divided in three subtypes: (i) conventional, (ii) chondroid, and (iii) dedifferentiated.

The morphology differs for each subtype. Conventional type is characterized for the presence of “physaliphorous cells” associated to myxoid matrix while biphasic tumors with irregular and high-grade undifferentiated sarcoma cells can be detected in dedifferentiated type. Matrix similar to the hyaline cartilage can be detected in chondroid type (116, 133, 134). The “poorly differentiated chordoma” is an entity characterized by the absence of SMARCB1 (i.e., INI1) expression originated from the skull base and cervical spine. This subtype has been described in pediatric patients and has been associated to poor clinical outcomes (134, 135). Differentiation between chordoma and chondrosarcoma is possible through the expression of a wide-spectrum of keratin in immunohistochemistry as well as expression of T brachyury in chordoma. Moreover, chordomas express cytokeratin, EMA, s1000 and presents physaliferous elements, which may help diagnosis.

About prognosis, dedifferentiated chordoma presents worse PFS and OS compared to classic subtype (136, 137). Indeed, 3-year PFS range from 40% in dedifferentiate chordoma to 85.6% in classic subtype and 100% in chondroid chordoma (136, 137). Similarly 3 years OS is 60% in dedifferentiated chordoma, 89.4% in chondroid chordoma and 100% in classic subtype (136, 137).

Management of chordoma of the skull base is difficult due to the proximity of these tumors to brain stem and major arteries (131, 136, 137). Available data seems to support the role of radical surgery followed by radiation therapy (especially when excision is incomplete) as best treatment approach (129, 135, 136). Few and not conclusive data about efficacy of systemic treatment in chordoma management are available. Kinases inhibitors have shown only modest benefit in pilot and phase II studies (129, 130, 135–138) thus shared opinion is that chemotherapy is not effective in these tumors.

Some trials are currently evaluating clinical efficacy of an EZH2 inhibitor named Tazemetostat in patients with poorly differentiated chordoma with SMARCB1 loss (NCI# NCT02601950 and NCT02601937) (138). However, due to the small number of patients enrolled and the short follow up available preliminary results and reports of efficacy are limited (138).

## Conclusion

Rare primary brain tumors in adults form a very large group of heterogeneous entities. Significant advances have been accomplished in diagnostic criteria thanks to the 2016 World Health Organization criteria and the unofficial, but broadly recognized amendments of the c-IMPACT-NOW Consortium. Standards of care are lacking for most rare adult primary brain tumors due to their scarcity. Thus, conducting robust clinical trials is very challenging. Joining forces in the setting of EURACAN Domain 10 and other neuro-oncology research networks will undoubtedly support, on the one hand, routine medical management of patients and, on the other hand, basic and clinical research toward improvement of prognosis for these patients.

## Author Contributions

EF, DF, RR, PH, and MP contributed conception and design of the study. EF, DF, RR, PH, CH, GL, SA, CD, and MP wrote the first draft of the manuscript. All authors wrote sections of the manuscript and contributed to manuscript revision, read, and approved the submitted version.

## Conflict of Interest

The authors declare that the research was conducted in the absence of any commercial or financial relationships that could be construed as a potential conflict of interest.
